# Deficit irrigation strategies (PRD, SDI) and titanium nanoparticles improve water use efficiency and flower quality in greenhouse-grown cut roses

**DOI:** 10.1038/s41598-023-45042-1

**Published:** 2023-10-21

**Authors:** Seyed Mohammad Alavi, Maryam Kamali, Yahya Selahvarzi, Sana Ansari

**Affiliations:** 1 Department of Water Science and Engineering, University of Tehran, Tehran, Iran; 2 Department of Horticultural Science, Faculty of Agriculture, Ferdowsi University of Mashhad, Mashhad, Iran

**Keywords:** Environmental impact, Nanoparticles, Plant physiology, Stomata, Drought, Plant signalling

## Abstract

This study explored the use of deficit irrigation techniques for water management in the hydroponic greenhouse cultivation of cut roses. A factorial experiment was conducted using three irrigation treatments: full irrigation (FI), partial root drying (PRD), and sustained deficit irrigation (SDI), and three doses of titanium dioxide nanoparticle foliar application (0, 15, and 30 ppm) as stress alleviation. Results showed that drought stress increased biochemical parameters such as the plants' proline and total phenol content. Compared to SDI treatment, the PRD treatments have an increase in flower number by 40%. The PRD strategy has positive effects on drought tolerance by increasing osmotic and elastic adjustment. Therefore, higher relative water content and longer root length in PRD treatments were observed. Thus, Biomass water use efficiency significantly increased in PRD treatments compared to others. In the PRD treatment, yield WUE increases by 26% and 61% compared to FI and SDI, respectively. The results showed TiO_2_-NPs positively affected mitigating and even improving some traits in drought stress conditions. These results suggest the superiority of the PRD strategy, which improves growth characteristics and water use efficiency, leading to increased sustainability, reduced environmental impact of greenhouse toxic wastewater, and total profitability of the greenhouse.

## Introduction

Water scarcity is a significant constraint for the agricultural industry on a global scale. To address this issue, greenhouse cultivation using soil and soilless substrates has been extensively adopted^1^. The agricultural sector faces a significant obstacle in the form of preserving and enhancing water usage efficiency, which can be attributed to climate change, inadequate water resources, and escalating expenses related to water supply that is appropriate for hydroponic farming. Additionally, the costs of fertilizer inputs, chemicals, and micronutrients have risen, further exacerbating the issue. The consideration of environmental policies and the sustainability of water resources is imperative in the context of this chain, as excessive irrigation has been found to result in reduced water use efficiency, increased CO_2_ emissions, and wastewater (total greenhouse unusable water discharge), according to Nikolaou et al. (2019)^1^.

Throughout history, roses have held significant cultural significance across various societies. Modern times, they are recognized as the primary contributor to the global cut flower industry^2,3^. Hydroponic farming utilizes less energy to absorb water and reduces the oxygen deficit^4,5^. The cultivation of cut-roses in a greenhouse setting is recognized as a plant that necessitates meticulous management of fertigation, involving substantial inputs of water, fertigation, and chemicals, as noted by Cabrera et al. (2009) and Franco-Hermida et al. (2020)^6,7^. The most prevalent hydroponic cultivation system is open-loop. The nutrient solution is not reused after it exits the system but is converted into wastewater. Fertigation is a commonly employed agricultural practice to mitigate plant water stress and nutrient deficiency. A high leaching proportion, between 20 and 50% ^4,5^, prevents salt accumulation in the plant's roots. According to Lizarraga et al. (2003), open-loop systems result in the loss of water and nutrients, leading to environmental issues^5^.

Estimating the irrigation dose was a major challenge. Despite numerous efforts to monitor and implement precise irrigation in greenhouses with artificial substrates, there is a gap between scientific and commercially viable solutions that farmers find appealing. Notably, farmers control a significant portion of the water and fertilizer consumption processes and efficiency in greenhouses. Mathematical models have been developed to improve water use efficiency, reduce environmental issues, and increase greenhouse productivity by reviewing irrigation resources for artificial substrates. They're not commercialized. Sensor-based models, the Internet of Things (IoT), and artificial intelligence-based image processing have also been used. Due to high set-up costs and complications, it has not reached economic use^1,8–10^. Empirical formulas calibrate for different plants and greenhouses based on environmental parameters inside or outside the greenhouses^4,11^. Moreover, some methods offered different formulations based on the characteristics of the substrate^12,13^; however, due to the limitations of these methods, estimating the amount of irrigation in greenhouses based on the knowledge of greenhouse owners and environmental conditions in the region remains the most prevalent and accepted method. Problems with these traditional approaches include inefficient use of water and nutrients and the production of wastewater that threatens the environment^1,14, 15^.

To address issues related to water scarcity, several deficit irrigation methods, such as sustained deficit irrigation (SDI), regulated deficit irrigation (RDI), and partial root-zone drying (PRD), are utilized as effective strategies for conserving water. It has been observed that PRD methodologies have yielded remarkable outcomes in terms of augmenting water usage efficiency while simultaneously ensuring minimal or negligible reductions in crop yield. This has been substantiated by various studies conducted by Sepaskhah and Ahmadi (2012)^16^, de Lima et al. (2015)^17^, and Marcelis and Heuvelink (2019)^18^, which have reported impressive findings in this regard. Compared to SDI conditions, this application method helps plants cope with drought stress and boost photosynthesis and yield^16,19^. Recent reports indicate that research on drought and salinity for cut roses is extremely limited and insufficient^2,20^.

The use of cutting-edge technology in hydroponic farming, such as nanoparticles, has become increasingly common to boost profitability and ensure long-term viability. TiO_2_-NPs are effective in various of plant biological processes, including photosynthesis, resistance to drought, and salinity, across several studies^21,22^. Several studies have been carried out in nanotechnology, revealing that different nanoparticles possess varying capacities as fertilizers, stress mitigators, and growth boosters. Titanium nanoparticles are an essential and extensively utilized type of particle. Prior research has indicated that these particles can mitigate the adverse impacts of water scarcity stress while simultaneously enhancing overall performance and productivity^23–27^. Nowadays, this emerging technology is combined with simple but effective strategies of deficit irrigation and has produced a very impressive output.

The main hypothesis of this research was based on over-irrigation and excessive drainage in hydroponic cut-flower roses. This problem can be addressed using the proper irrigation strategies and some stress alleviators. Over the past decades, these strategies have been used in farms, orchards, and soil cultivation greenhouses. Still, the hydroponic research upon this methods is very limited especially in ornamental plants. Therefore, this study aims to increase water productivity in hydroponic roses cultivation by implementing partial root-zone drying (PRD) and sustained deficit irrigation (SDI) techniques. Nanotechnology, such as TiO_2_-NPs foliar spraying is also employed to ameliorate the adverse stress and improve the flower quality of plants.

## Material and methods

### Plant and greenhouse condition

**Figure 1:**
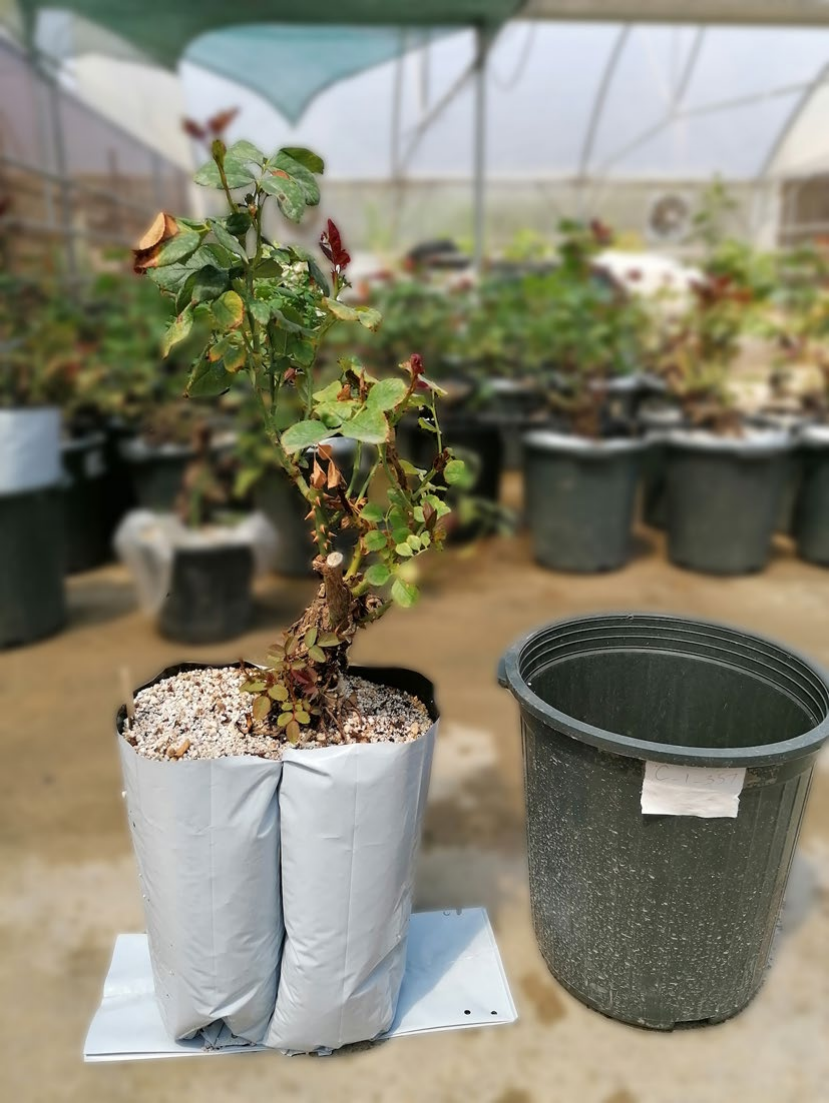
Applying the treatment and placing it in the pot is specified.

This project was carried out in a research greenhouse at the University of Ferdowsi, Mashhad, Iran (36.29β° N, 59.60° E) in the summer of 2020 in a factorial experiment based on completely randomized design with four replications on *Rosa hybrid*, L. cv. Red one. The cultivar was grafted onto *Rosa hybrid* L. ‘Natal Briar’ rootstock, purchased from the Special Horticulture Unit of Ferdowsi University of Mashhad. This experiment has been implemented with one flowering flash. The greenhouse ventilation system was a fan-pad ventilation system, and the light intensity was controlled using a shade. The minimum and maximum temperature and humidity data were recorded thrice daily. Average temperature of 20/28 ^°C^ Day/Night and relative humidity of 60% were adjusted with the fan-pad ventilation system. Moreover, we used artificial light to provide the 14/10 h day/night regime. Two-year-old plants were transferred to the research greenhouse after preparation on April 23 (Fig. 1). Initial pruning was performed, and the experiment continued for five months. Two weeks of planting time and fertilizer application are considered for all pots without any water deficit. Irrigation was performed with “Hoagland’s nutrient solution” twice a week to provide plant nutrient needs. A drip system applied irrigation with two emitters for each pot. Daily irrigation cycle was implemented at 08:00 PM, 12:00 PM, and 04:00 AM for four months. The experiment is composed of four replications for each treatment and each replication is made of three plants; in total 108 pots were used. The collection of plants material complies with relevant institutional, national and international guidelines and legislation and permission was obtained for the collection of the plant material.

### Treatments

The first treatment was four water management strategies applied after plants consolidation. In FI, the whole pot capacity (FC) was equally given in both grow bags; SDI treatments were implemented the same way as FI with half amount of pot capacity; In PRD1, 50% of Fl was applied in one grow bag, and the other grow bags stayed dry for three irrigations; In PRD2, the grow bags altered every irrigation. The second treatment was applied two weeks before the irrigation treatments, and foliar application of nano titanium dioxide (NP- TiO_2_) with three concentrations (0, 15, and 30 ppm) was used four times at seven-day intervals. A German company named Evonik Degussa GmbH prepared the titanium dioxide nanoparticles that were used. The average particle size was 21 nm and with a purity of 99.5%. An ultra-sonication treatment was applied to TiO_2_-NPs powders dispersed in water for 15 min to obtain properly stable NP- TiO_2_ suspensions of each concentration. At each time, 300cc of a solution containing different titanium dioxide nanoparticles was sprayed onto the plant air surface.

### Irrigation control

In this project, an open irrigation system was used, automatically irrigating three times a day based on a digital timer. Pressurized drippers and diaphragm pumps were used for accurate irrigation. Water irrigation qualities maintained in pH 5.5 to 6.0, EC (d/Sm^−1^) 1.5–2.5 suitable for roses^28^. We determined daily irrigation volume (V) based on common irrigation in commercial greenhouses in the region and Eq. (1). The V represents the volume of water for irrigation in meters, Epan represents the evaporation rate from a Class A evaporation pan within the greenhouse in millimeters, and SA represents the shaded area in square meters. The shading level is determined by multiplying the distance between pots in each row by the row spacing.

1 $$V=(1000 \times \mathrm{Epan})(SA)$$

The volume of irrigation water in this experiment was 95 (liters/pot) for FI treatment and for other treatments were 50% of this amount. Therefore, using the method provided by Mavrogianopoulos (2016), based on the characteristics of the substrates and knowing the water retention curve of the substrate, pot water holding capacity, Irrigation dose and irrigation duration were estimated^13^. Subsequently, two thresholds were established in PRD-1 and PRD-2 based on the availability of easily accessible water (EAW) and a leaching fraction of 20%. These thresholds served as the determining factor for when irrigation should be shifted from one side to the other.

### Measurements

#### Harvest and plant fresh and dry matter

**Figure 2:**
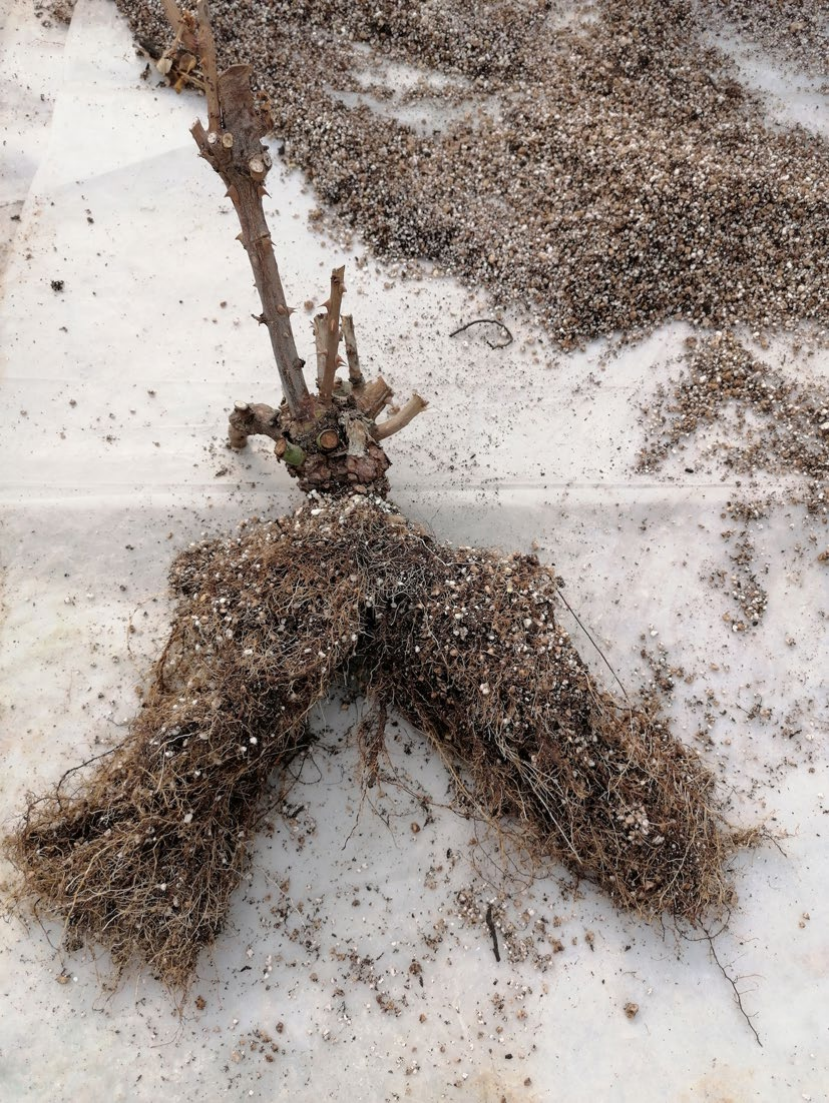
Separated roots at the end of the plot.

After 60 days from the start of treatment, physical characteristics of the plant such as the diameter of flowers, length, the diameter of flowering stems, and the number of leaves were recorded. The total leaf area is calculated by leaf area meter (Li-cor 1300, USA). Dry weight (DW) of aerial parts and roots were weighted separately for the left and right side of the plant using a digital scale (GF, 300) with an accuracy of 0.001 kg (Fig. 2). Each side root length was measured after removing all substrates and washing them carefully with a ruler.

#### Analysis of plant compounds and properties

Antioxidant activity (AA) was measured using the DPPH method^29^. Total phenolic compounds (TP) were determined using a UV/V spectrophotometer (Jenway, Model 6305) at 660 nm using Folin–Ciocalteu as a reagent. Results were reported as milligrams (mg) of gallic acid per 100 ml^30^. Total anthocyanin content was analyzed using a pH differential method with two buffer systems. The pH meter (Elmetron CP-501) was used for this experiment. Sodium acetate and potassium chloride by pH 4.5 and 1.0 were used the method^31^. Total flavonoids were measured by the aluminum chloride (AlCl_3_) colorimetric method using quercetin as standard. Results were expressed in mg of quercetin per gram of plant extract^32^. Free proline was extracted from 50 mL of cell sap with sulfosalicylic acid (3%) and quantified according to the protocol of Bates et al. (1973). Total carbohydrate content was extracted from the dried plant material and determined spectrophotometrically at 640 nm using the method of Staub (1963). Relative water content (RWC) was measured by random sampling of plants' last healthy middle leaf at 10 a.m., based on Shahrivar et al. (2020) method, and iron leakage was calculated based on Karlidag et al. (2009) suggested approach. Extraction and assay of chlorophylls b, a, total chlorophyll and leaf carotenoids were performed using the Lichtenthaler and Wellburn method (1983)^33^.

#### Water use efficiency (WUE)

Biomass WUE was considered the ratio of cumulative aboveground dry weight (g) to evapotranspiration (ET) used by the plant (kg or cm^1^). The ratio of product dry mass (g) to ET is yield WUE. Finally, the effective WUE (WUE^1^) was the yield of dry-weight biomass (g) to the total water consumption in the greenhouse per pot. For practical purposes, WUE^1^ is better than WUE biomass because the leaching fraction included in the concept is relatively high in hydroponic greenhouses^34^.

#### Measurement of photosynthetic efficiency

We use a portable LCA4 device to measure the photosynthesis rate (carbon dioxide assimilation)(A), transpiration rate (E), and stomatal conductance (gs). We used the plant's young and fully opened leaves in a non-destructive manner to calculate these parameters. We measured four digits from each plant and reported the average as the final number for each treatment. The measurement happened from 9:00 a.m. to 12:00 p.m. in the final week of the experiment.

#### Catalase (CAT) and ascorbate peroxidase (APX)

Catalase activity was specified by monitoring the disappearance of H_2_O_2_ by spectrophotometry according to the method of Cakmak and Marschner Amira^35^. The activity of ascorbic acid peroxidase was specified by spectrophotometry by measuring the absorbance at 290 nm and estimating the consumption of the ascorbate substrate. One unit of APX activity was defined as the enzyme required to consume 1 mol of ascorbate per minute^35^.

#### Determination of nutrient elements (K, P, Mg, Zn, and Ti)

The ICP-OES was calibrated with the standard solution for the various elements. As the solid samples cannot be submitted directly into the plasma, they must either be ablated by laser or made into a solution for ICP-OES analysis of the target or main elements. The microwave digestion technique was employed to prepare the plant samples in liquid form. For analysis of the rose plant samples by ICP-OES, about 0.0265−0.0278 g/100 ml of each sample was dissolved in 10% HNO_3_ and 1% HCl, then heated for about 1 h at 120 ºC to reach complete dissolution of the samples. Finally, after proper dilution, the prepared liquid samples were inserted into the PerkinElmer Optima 5300 DV ICP-OES (PerkinElmer, Inc, USA) for the elemental analysis^36^.

### Statistical analysis

The collected data were analyzed using Jump software (JMP 8) with the least significant difference (LSD) test at 5% probability.

## Results

### Accumulation of nutrients

The uptake and accumulation of nutrients (in shoots) including phosphorus (P), potassium (K), zinc (Zn), magnesium (Mg), and titanium (Ti) were also observed in this experiment (Table 1). Different irrigation regimes influenced the accumulation of P, K, Zn, and Mg (*P* ≤ 0.05), as well as Ti (*P* ≤ 0.01). Under SDI (50% FC), nutrient accumulation (K, P, Mg, and Zn) was decreased in the shoots. So, K, P, Zn, and Mg accumulation showed the lowest values in the SDI treatment. TiO_2_-NPs gave significant results in the sole and were combined with different levels of deficit irrigation. The application of 30 ppm TiO_2_-NPs increased the levels of P, K, Zn, Mg, and Ti to 6.5%, 9.5%, 6.5%, 0.7%, and 32%, as compared to the non-foliar application of TiO_2_-NPs in 100% FC. Foliar application of 30 ppm TiO_2_-NPs enhanced the content of K to 10.7 and 28.9%, Zn to 50.36 and 42.25%, and Ti to 44.51 and 39.33% as compared to the non-foliar application of TiO_2_ in PRD1 and PRD2, respectively. The highest values of Mg content were measured in the rose leaves in PRD2 + 30 ppm TiO_2_-NPs (3854 ppm). Regarding P accumulation, the values ranged between 1123 and 1564 ppm in the studied plants. It was noticed that P accumulation reached 1564 ppm in PRD2 + 15 ppm TiO_2_-NPs.

**Table 1:** The interaction effect of deficit irrigation × TiO_2_-NPs on some mineral accumulation in rose plants under deficit irrigation.

Treatments		K (ppm)	P (ppm)	Mg (ppm)	Zn (ppm)	Ti (ppm)
Deficit irrigation	TiO_2_-NP (ppm)					
Control (FI)	30	7573.50^a^*	1475.94^ab^	2159.59^bc^	18.36^ cd^	66.54^bc^
	15	6885.40^ab^	1495.66^ab^	2644.40^bc^	30.12^a^	39.945^e^
	0	6917.23^ab^	1384.80^abc^	2144.39^bc^	17.23^ cd^	50.37^cde^
PRD1	30	7811.01^a^	1327.46^bcd^	2606.91^bc^	26.54^abc^	96.19^a^
	15	6639.61^bc^	1387.36^abc^	2740.10^bc^	28.03^ab^	68.25^b^
	0	7056.10^ab^	1435.40^abc^	2909.62^b^	17.65^ cd^	66.56^bc^
PRD2	30	7662.47^a^	1482.21^ab^	3854.24^a^	19.83^bcd^	69.43^b^
	15	7755.11^a^	1564.99^a^	2123.14^bc^	19.38^bcd^	47.04^de^
	0	5944.42^c^	1156.32^de^	2449.25^bc^	13.94^d^	49.83^de^
SDI	30	6379.59^bc^	1456.62^ab^	2213.93^bc^	15.41^d^	62.36^bcd^
	15	6287.42^bc^	1123.73^e^	1942.87^c^	17.69^ cd^	57.85^bcd^
	0	6634.46^bc^	1252.52^cde^	2457.64^bc^	14.49^d^	58.11^bcd^

*Means followed by same letters in each column does not have a significant difference based on the LSD test (*p* ≤ *0.01*).

### Biochemical and physiological parameters (electrolyte leakage (EL), relative water content (RWC), antioxidant activity (AA), total phenol (TP), flavonoid content (FC), total soluble carbohydrates (TC), and proline content (PC))

The shoots' flavonoid content and total soluble carbohydrates were affected by deficit irrigation and TiO_2_-NPs application (P ≤ 0.01) and not by their interaction. The use of TiO_2_-NPs led to an increase in flavonoid content and total soluble carbohydrates in shoots. As the amount of TiO_2_-NPs fertilizer increased, the content of these compatible osmolytes in the shoots also increased. Using TiO_2_-NPs at 30 ppm induced compatible osmolytes 26.1 and 34.55% more than the control for flavonoid content and total soluble carbohydrates, respectively (Table 2B). Also, these compound contents increased when deficit irrigation was applied. SDI-treated plants had 36.44% and 23.71% more flavonoid content and total soluble carbohydrates than non-deficit irrigated plants (Table 2B). The relative water content was affected by deficit irrigation; that is, the treatment with PRD1 and PRD2 enhanced relative water content, and the SDI treatment had the lowest relative water content of 72.9%. ANOVA revealed that traits regarding physiological and biochemical content, including electrolyte leakage, proline content, antioxidant activity, and total phenol were significant under the interaction of deficit irrigation and various levels of TiO_2_-NPs. The electrolyte leakage measured in leaves treated with the TiO_2_-NPs was the lowest across all irrigation levels.

**Table 2:** The interaction effect of deficit irrigation × TiO_2_-NPs (A) and simple effect of them (B) on biochemical and physiological parameters of the rose plant.

(A)						(B)				
Deficit irrigation	TiO_2_-NP (ppm)	EL (%)	PC (mg/gfw)	AA (%)	TP (eq galic acid/1gr dw)			RWC (%)	FC (eq quersetin/1gr dw)	TC (mg/gdw)
FI	30	19.24^ef^*	11.82^bcd^	78.56^abc^	27.50^bcd^					
	15	26.60^c^	12.80^bc^	81.15^ab^	30.46^ab^	Deficit irrigation	FI	73.69^bc^	4.39^b^	152.37^b^
	0	25.98^ cd^	6.49f.	75.39^bc^	28.36^bc^		PRD1	79.46^a^	4.66^b^	119.60^c^
							PRD2	77.00^ab^	4.89^b^	141.20^bc^
PRD1	30	18.81f.	7.38f.	78.13^abc^	22.66^ef^		SDI	72.90^c^	5.99^a^	188.44^a^
	15	18.92f.	6.95f.	78.56^abc^	26.23^cde^					
	0	32.15^b^	7.47f.	71.71^c^	21.98f.					
PRD2	30	24.07^c−f^	13.06^b^	80.39^ab^	27.15^bcd^	TiO_2_-NP (ppm)	30	–	5.60^a^	174.29^a^
	15	21.28^def^	7.82^ef^	76.02^bc^	29.26^abc^		15	–	4.91^b^	147.38^b^
	0	28.45^bc^	7.08^ef^	62.57^d^	20.36f.		0	–	4.44^b^	129.53^b^
SDI	30	24.38^cde^	10.59^ cd^	80.17^ab^	33.10^a^					
	15	24.12^c−f^	15.59^a^	84.36^a^	30.85^ab^					
	0	40.74^a^	10.02^de^	79.39^ab^	24.14^def^					

*Means followed by same letters in each column does not have a significant difference based on the LSD test (*p* ≤ *0.01*).

Moreover, the electrolyte leakage was maximum in SDI treatment without TiO2-NPs application; a downward trend in electrolyte leakage with increasing TiO_2_-NPs concentration for all irrigation levels was observed. The mean electrolyte leakage was in the range of 18.81–40.74% (Table 2A). When the irrigation was in the form of SDI, leaves showed the highest proline content (15.59 mg g^−1^fw) and antioxidant activity (84%) with the application of TiO_2_-NPs at 15 ppm (Table 2A). Leaves could produce higher levels of phenolic compounds when sprayed with nanoparticles, especially in plants treated with the SDI form of deficit irrigation. The highest values for total phenol (33.1eq gallic acid. gr^−1^ DW) were determined for plants treated with SDI + 30 ppm TiO_2_-NPs (Table 2A).

### Antioxidant enzymes activity (Ascorbate peroxidase (APX) and catalase activities (CAT)):

The activities of APX and CAT in rose leaves, were determined under the different experimental treatments (*P* ≤ 0.01). The lowest activities of APX and CAT were observed in control conditions. Moreover, the application of TiO_2_-NPs was shown to be an effective way to increase enzymatic antioxidant activities (Table 3). The highest activity of CAT was observed in SDI with the application of 30 ppm TiO_2_-NPs (75.32 μmol H_2_O_2_ mg^−1^ protein. min^−1^). Leave APX activity was higher in PRD1 + 15 ppm TiO_2_-NPs and SDI + 30 ppm TiO_2_-NPs than other treatments.

**Table 3:** The interaction effect of deficit irrigation × TiO_2_-NPs on antioxidant enzymes activity (Ascorbate peroxidase (APX) and catalase activities (CAT)):

Deficit irrigation	TiO_2_-NP (ppm)	Catalase (μmol H_2_O_2_ mg^−1^ protein min^−1^)	Ascorbate peroxidase (unit. g^−^1fw)
Cont. (100% FC)	0	60.69^ef^*	3.73^e^
	15	67.34^bc^	4.72^ cd^
	30	66.68^bcd^	4.41^d^
PRD1	0	57.48f.	5.17^bc^
	15	65.44^cde^	5.71^a^
	30	69.99^abc^	5.12^bc^
PRD2	0	61.28^def^	5.26^ab^
	15	58.02f.	5.53^ab^
	30	70.21^abc^	5.08^bc^
SDI	0	66.50^bcd^	5.57^ab^
	15	71.77^ab^	5.48^ab^
	30	75.32^a^	5.76^a^

*Means followed by same letters in each column does not have a significant difference based on the LSD test (*p* ≤ *0.01*).

### Photosynthetic pigments and carotenoid content

We evaluated the effect of TiO_2_-NPs and irrigation methods on transpiration (Tr), stomatal conductivity (Gs), and photosynthesis activity (P) in roses. Adding TiO_2_-NPs dramatically enhanced Tr, Gs, and P compared with a TiO_2_-NPs treatment (no spray with TiO_2_-NPs) (Table 4A and B). Also, SDI treatment sharply decreased Tr and Gs in roses at all levels of TiO_2_-NPs and the P in leaves. PRD methods markedly improved Tr, Gs, and P of roses; the highest transpiration was observed in PRD2 + 30 ppm NPs, and the highest stomatal conductivity was observed in PRD2 + 10 or 20 ppm TiO_2_-NPs. Furthermore, ANOVA revealed that the studied interaction was statistically significant in terms of chlorophyll-a (*p* < 0.05), chlorophyll-b (*p* < 0.01), and total chlorophyll (p < 0.01). The highest values for chlorophyll b (6.259 µg/ml) and total (12.669 µg/ml) were determined for plants treated with PRD1 + 30 ppm TiO_2_-NPs (Table 4A). Leaves could produce a higher chlorophyll-a content when treated with 15 ppm nanoparticles + PRD2 and 30 ppm nanoparticles + PRD1. Deficit irrigation and TiO_2_-NPs had a significant effect on carotenoid content as a simple effect; that is the increase in TiO_2_-NPs levels led to enhancement of the carotenoid content, and plants without being treated with TiO_2_-NPs had the least carotenoid content (Table 4B). PRD1 and PRD2 induced the highest carotenoid content in the leaves, while the lowest amount was detected by SDI and control treatments.

**Table 4:** The interaction effect of deficit irrigation × TiO_2_-NPs (A) and simple effect of them (B) on gas exchange and photosynthetic pigments of the rose plant.

(A)							(B)			
Deficit irrigation	TiO_2_-NP (ppm)	Chlorophyll a (mg g^−1^ fw)	Chlorophyll b (mg g^−1^ fw)	Total chlorophyll (mg.g^−1^ fw)	Transpiration (mmol H2O.m^−2^.S^−1^)	Stomatal conductance (mol CO2.m^−2^.S^−1^)			Carotenoid (mg g^−1^ fw)	Photosynthesis (µmol CO_2_ m^−2^ S^−1^)
FI	0	4.758^bcd^*	3.898^ cd^	8.656^ef^	0.54^ef^	0.46^ef^				
	15	4.796^bcd^	4.639^bcd^	9.435^c−f^	0.78^bc^	0.65^ cd^	Deficit irrigation	FI	0.151^b^	17.33^b^
	30	4.876^bcd^	4.308^ cd^	9.184^c−f^	0.76^bcd^	0.64^cde^		PRD1	0.229^a^	18.93^ab^
								PRD2	0.217^a^	19.25^a^
PRD1	0	4.853^bcd^	4.589^bcd^	10.984^b^	0.78^bc^	0.81^abc^		SDI	0.132^b^	14.85^c^
	15	5.710^ab^	4.141^ cd^	10.477^bc^	0.88^ab^	0.78^bc^				
	30	6.408^a^	6.259^a^	10.335^bcd^	0.87^ab^	0.75^c^				
PRD2	0	5.067^bc^	4.287^ cd^	10.298^bcd^	0.56^ef^	0.49^def^		30	0.191^b^	19.96^a^
	15	6.696^a^	5.410^ab^	8.993^def^	1.04^a^	0.99^a^	TiO_2_-NP (ppm)	15	0.248^a^	19.79^a^
	30	5.679^ab^	4.657^bc^	12.667^a^	1.02^a^	0.95^ab^		0	0.107^c^	13.02^b^
SDI	0	4.382^ cd^	3.760^d^	8.141f.	0.37f.	0.32f.				
	15	4.390^ cd^	5.297^b^	9.687^b−e^	0.63^cde^	0.50^def^				
	30	3.917^d^	4.696^bc^	8.613^ef^	0.58^de^	0.51^de^				

*Means followed by same letters in each column does not have a significant difference based on the LSD test (*p* ≤ *0.01*).

### Vegetative traits

The interaction of deficit irrigation × TiO_2_-NPs significantly affected vegetative traits, including root dry weight, leaf area, and root length (*P* ≤ 0.01). Rose plants had the highest root dry weight (52.84 g) and root length (85.00 cm) when grown using 30 ppm of TiO_2_-NPs and treated with PRD1 and PRD2, respectively; plants without TiO_2_-NPs fertilizing and treated with SDI showed the lowest root dry weight (25.4g) (Table 5). In deficit irrigation treatments, the leaf area was affected by TiO_2_-NPs in a positive trend. The leaf area increased to 564,752 cm^2^ in PRD2 + 30 ppm TiO_2_-NPs, while the leaf area for non-TiO_2_-NPs plants with control irrigation was 278,039 mm^2^. The shoot dry weight increased by TiO_2_-NPs application, whereas the lowest shoot dry weight (38.91 g) was recorded in non-TiO_2_-NPs application. On the other hand, the highest amount of dry shoot weight (51.61 g) was observed in PRD1 irrigation (Table 6).

**Table 5:** The interaction effect of deficit irrigation and TiO_2_-NPs on growth attributes of the rose plant.

Deficit irrigation	TiO_2_-NP (ppm)	Root dry weight (g)	Flower dry weight (g)	Leaf area (mm^2^)	Root length (cm)
Cont	0	34.30^c^*	1.72^a^	278039f.	52.75^b^
(100% FC)	15	42.32^b^	1.38^ab^	288484f.	56.15^b^
	30	29.26^cde^	1.35^ab^	406601^cde^	57.75^b^
PRD1	0	31.34^cde^	1.68^a^	355340^ef^	55.50^b^
	15	42.88^b^	1.44^ab^	505412^ab^	53.50^b^
	30	52.84^a^	1.57^ab^	457532^bcd^	52.00^b^
PRD2	0	30.46^cde^	1.39^ab^	479043^abc^	85.00^a^
	15	33.25^ cd^	1.70^a^	363388^def^	61.25^b^
	30	34.53^c^	1.41^ab^	564752^a^	56.75^b^
SDI	0	25.48^e^	1.42^ab^	166264^ g^	60.75^b^
	15	30.57^cde^	1.41^ab^	291551f.	55.75^b^
	30	27.54^de^	1.17^b^	316965^ef^	51.50^b^

*Means followed by same letters in each column does not have a significant difference based on the LSD test (*p* ≤ *0.01*).

**Table 6:** The simple effect of deficit irrigation and TiO_2_-NPs floral and vegetative traits of the rose plant.

		Flower number	Shoot dry weight (g)	Peduncle diameter (mm)	Flowering stem length (cm)	Flower diameter (mm)	Anthocyanin content (mg/g. dw)
Deficit irrigation	Cont. (100% FC)	2.66^ab^*	37.800^c^	3.22^b^	48.06^b^	72.50^b^	6.47^b^
	PRD1	3.33^a^	51.618^a^	3.51^a^	53.64^ab^	80.76^a^	8.10^a^
	PRD2	3.33^a^	45.296^b^	3.34^ab^	54.06^a^	81.83^a^	7.42^a^
	SDI	2.00^b^	33.104^d^	3.14^b^	41.20^c^	75.87^b^	5.99^b^
TiO_2_-NP (ppm)	30	3.50^a^	38.919^b^	3.53^a^	48.19^ab^	81.37^a^	6.57^b^
	15	2.75^ab^	43.042^a^	3.28^b^	52.92^a^	77.26^b^	7.93^a^
	0	2.25^b^	43.903^a^	3.09^b^	46.60^b^	74.59^b^	6.49^b^

*Means followed by same letters in each column does not have a significant difference based on the LSD test (*p* ≤ *0.01*).

### Floral traits

The flower dry weight was affected by the interaction of deficit irrigation × TiO_2_-NPs (Table 5). In contrast, the flower number, peduncle diameter, flowering stem length, flower diameter, and petal anthocyanin content were not influenced by interaction but were affected by their simple effect (*P* ≤ 0.01) (Table 6). The plants grown under SDI treatment without TiO_2_-NPs (1.17 g) had the lowest flower dry weight (Table 5). We obtained the highest peduncle diameters (3.51 and 3.34 mm) in PRD1 and PRD2 treatments, respectively (Table 6). The flower number had the highest value (3.33) when treated plants with PRD1 and PRD2, and the lowest value (2) was recorded in the SDI irrigation. Rose plants had the highest flowering stem length (53.64 and 54.06 cm), flower diameter (80.76 and 81.83 mm), and anthocyanin content (8.10 and 7.42 mg/g DW) when grown under PRD1 and PRD2, while plants treated with SDI showed the lowest flowering stem length (41.2 cm). The flower diameter and anthocyanin content had the lowest values in the control and SDI treatments without significant differences. The floral traits were affected by TiO_2_-NPs; that is, the increase in TiO_2_-NPs levels led to enhancement of the floral traits, and non-spraying plants produced fewer flowers and less peduncle diameter, flowering stem length, and flower diameter. So, the highest value was recorded on plants treated with 30 ppm of TiO_2_-NPs: 3.50, 3.53 mm, 48.19 cm, and 81.37 mm, respectively. The application of 15 ppm TiO_2_-NPs induced the highest anthocyanin content (7.93 mg/g DW), while the lowest amount was detected when no TiO_2_-NPs fertilizer was used or in 30 ppm TiO_2_-NPs (Table 6).

### Water use efficiency

Yield WUE was increased in PRD treatment compared to SDI and FI; SDI has the lowest yield WUE (0.028 g/kg) in general, although there was no significant difference in PRD and FI treatments (Table 7). The PRD strategies had a significant increase in biomass WUE; PRD1 showed the highest (0.72 g/kg) and SDI the lowest (0.35); also, the simple act of applying TiO_2_-NPs increased the biomass WUE significantly (Table 7). Moreover, WUE^1^ had a decrease in TiO_2_-NPs treatments; meanwhile, PRD applications had a higher increase in WUE^1^; the lowest record was 0.028 in SDI treatments; in contrast, PRD treatments have significantly increased in contrast to SDI treatments; the highest record was observed in the PRD1 with 0.056 (Table 7).

**Table 7:** The simple effect of deficit irrigation and TiO_2_-NPs on yield and water use efficiency.

		WUE^1^ (gr/kg)	Yield WUE (gr/kg)	Biomass WUE (gr/kg)
Deficit irrigation	FI	0.043^ab^*	0.054^a^	0.50^c^
	PRD1	0.056^a^	0.073^a^	0.72^a^
	PRD2	0.053^a^	0.071^a^	0.63^b^
	SDI	0.028^b^	0.028^b^	0.35^d^
TiO_2_-NP (ppm)	0	0.058^a^	–	0.51^b^
	15	0.044^ab^	–	0.56^a^
	30	0.034^b^	–	0.57^a^

*Means followed by same letters in each column does not have a significant difference based on the LSD test (*p* ≤ *0.01*).

## Discussion

To maintain rose production and water productivity, the rose plant must maintain a soil–plant-atmosphere water balance due to its low water storage capacity and high economic value^20^. The results showed encouraging outcomes from combining PRD strategies and TiO_2_-NPs technology to reduce adverse drought effects in roses.

In our study, we observed a higher concentration of Ti in the 30ppm TiO_2_-NPs treatments (Table 1). The observed phenomenon can be attributed to the notable characteristics of nanoparticles (NPs), including their elevated specific surface area, relatively high reactivity, and diminutive size (less than 100 nm)^22^. These properties facilitate the NPs' ability to readily infiltrate non-selective leaf uptake mechanisms, including the symplast pathway ^37,38^. Applying PRD2 treatments with TiO_2_-NPs resulted in enhanced water stress adaptation, as evidenced by improvements in transpiration and stomatal regulation, as shown in Table 4. It has been reported to increase photosynthetic efficiency, produce more chloroplasts, and deal with drought stress problems through the foliar application of titanium dioxide nanoparticles^26,39^. We also observed significantly higher photosynthesis rates in the presence of TiO_2_-NPs (Table 4). Naturally, its effect differs from one plant species to another ^40^.

Better absorption of elements by NPs has been reported in previous studies^40,41^. Our results show that despite the adverse effects of severe drought stress (SDI) on mineral accumulation in leaves, we have better absorption of elements in PRD treatments, especially in PRD2, as well as the positive effect of TiO_2_-NPs foliar application in all treatments (Table 1); this may be due to more use and absorption of mineral elements to cope with mild drought stress as a defensive reaction^42^. Potassium plays a key role in regulating membrane potential and helps regulate cell osmotic adjustment and ROS regulation. More K absorption by plants in drought conditions has been reported^43,44^; likewise, a recent study demonstrates an increase in K accumulation in roses under drought stress^45^. Despite no significant difference, we observed the highest K in PRD1 (Table 1). Root development is strongly associated with the absorption of nutrients, especially P^46^; PRD treatment and TiO_2_-NPs positively affect the accumulation of these elements (Table 1). TiO_2_-NPs have been reported beneficial in accumulating minerals such as N, P, K, Zn, and Cu^41,47^. They also reported increased N, P, calcium, and Mg uptake in TiO_2_-NPs foliar application on greenhouse tomato cultivation fields^48^. Our results (Table 1) indicate a positive correlation between an increased TiO_2_ dose and the accumulation of minerals. A recent study reported a positive effect of titanium on plant biomass, fruit quality, and Ca, Mg, P, iron, N, and Ti accumulation^37^.

Under drought conditions, restricting vegetative growth to limit transpiration levels is one of the primary defense responses of the plant^49,50^. The literature also states that the PRD technique causes more abscisic acid (ABA) secretion in roots; it sends a message to close or limit the diameter of the stomata, which prevents excess water from escaping through the leaves^51,52^. Our study observed that this stomata regulation happened in our PRD treatments (Table 4). Also, plants’ responses to PRD were better than those to SDI treatments (Tables 2 and 5). Better adaptability in this method can be due to the deeper wetting front in the root zone of roses. This means that we have a larger moisture layer with a certain amount of water; this phenomenon leads to more roots growing. Applying the PRD technique resulted in heavier and longer roots in our study compared to full irrigation and SDI treatments (Table 5); similar results are reported in other research, too^52–54^. Studies have reported that exposing the roots to a dry–wet irrigation cycle increases the roots hydraulic conductivity. This means the amount of water the plants requires is provided by the roots from the wet-side^52,55^. On the other hand, the risk of oxygen shortage on the wet side may be eliminated at full pot capacity^34^.

Leaf relative water content (RWC) is one of the main parameters used to evaluate the amount of gas exchange through the leaves^56^. A reduction in RWC means the plant encounters more abiotic stresses, such as drought. Regulation of leaf stomata is the plant’s reaction to drought stress that leads to reduced water and carbon dioxide exchanges, resulting in a reduced photosynthesis rate in the plants^57,58^. In our results, RWC in PRD1 treatment was significantly better than FI. Besides, SDI treatment was at the lowest level (Table 2), showing the highest stress level and the lowest performance and biomass WUE (Table 7). Drought stress reduces the water movement gradient in the soil–plant-atmosphere continuum to retain more water in the plants. This change is due to an important mechanism of plant adaptation to drought stress that changes the cell wall and the water potential inside the cells, leading to more water absorption from the roots and aerial parts of the plant^45,59^. A study reports that TiO_2_-NPs positively impacts RWC despite a higher stomatal conductance^60^. We observed a significantly higher photosynthesis rate in PRD irrigation treatments (Table 4), the same as the above reports.

In this study, we observed that TiO_2_-NPs improved plant tolerance mechanisms by upregulating antioxidant activity and modulating osmolyte levels in cells by increasing compatible organic osmolytes such as proline and sugar (Table 2), which are involved in stabilizing the cell wall and scavenging ROS^38^. Electrical leakage (EL) can be accelerated by a rise in ROS because it leads to increased cell destruction ^38,61^. Our results indicate the highest EL was observed in SDI treatment without TiO_2_-NPs, and TiO_2_-NPs foliar application reduced EL (Table 2). Prior research shows that more proline in the plant indicates both resistance and stress conditions, which depend on the plant species^53^. Considering the reports of a negative relationship between proline and RWC in ornamental plants^53^, *Damask rose*^45^, and the results of the present research (Table 2), it can be concluded that higher proline levels suggest more stress in the roses, as we observed a 35% increase in SDI compared to the control treatment (FI without TiO_2_-NPs); a lower amount of proline was reported in the control and PRD treatments, and the highest amount of proline was observed in SDI (Table 2).

It has been reported that severe dehydration led to a significant increase in plant phenol content in roses^62^, Pan American, and Cinderella^63^. Phenolic compounds protect the chlorophyll, with a positive relationship between phenolic content and AA reported in Hessini et al. (2022) research^62^, which can be seen in our results (Table 2). Moreover, in our experiments, increasing drought stress and the amount of titanium positively affected on the amount of total phenol in the plant (Table 2). The increase in flavonoids was related to the concentration of TiO_2_-NPs; the highest level was observed at 30 ppm (Table 2).

Antioxidant enzymes are increased in drought conditions to maintain ROS balance and control the negative effects of drought by modulating the pathways of genetic, biochemical, and physiological activities^53,64,65^. We observed an upward trend in CAT and APX in response to water shortage conditions (Table 3). Also, we observed that foliar application of TiO_2_-NPs increased the activity of antioxidant enzymes such as CAT and APX in the most severe drought conditions, the SDI treatments, in the plant (Table 3); similar results were reported on cotton (*Gossypium barbadense* L.)^39^, dragon's head (*Lallemantia iberica* L.)^23^, and wheat (*Triticum aestivum* L.)^60^. Moreover, research has reported a special protective role against oxidative stress, which has been attributed to the ability of nanoparticles to mimic the role of enzymes such as catalase, superoxidase, and superoxidase dismutase^66^; other studies have reported the same mechanism and alleviating role of TiO_2_ in drought stress^22^.

Previous research on roses documented that as drought stress intensifies in C3 plants, a reduction in plant greenness occurs due diminished chlorophyll levels^49^. Furthermore, we have the lowest total chlorophyll content in SDI treatments. In contrast, PRD treatments generally lead to better results; the highest total chlorophyll content with a 31% increase was observed in PRD1 + 30 ppm TiO_2_-NPs (Table 4A). Previous research has shown that TiO_2_-NPs can decelerate chloroplast aging^67^ and increase chlorophyll and carotenoids in plants^25,68, 69^. Also, a direct correlation between photosynthesis rate and chlorophyll has been reported^49^. This correlation can be seen in our results, where the highest total chlorophyll and photosynthesis results belong to PRD2 treatments (Table 4A and B). Additionally, The PRD1 treatment increased in carotenoid content by 34% and 42%, compared with FI and SDI, respectively (Table 4B).

TiO_2_-NPs were also found to increase biomass under drought stress in plants such as dragonhead plants *(Dracocephalum moldavia* L*.)*^38^, flax (*Linum usitatissimum* L.), and cotton (*Gossypium hirsutum* L.)^39^. It has been reported that using TiO_2_ to enlarge plant cells ultimately leads to a greater increase in biomass ^38^. Similar to our results, we have increased dry root weight by 47% in PRD1 + 30 ppm TiO_2_-NPs and shoot dry weight by TiO_2_-NPs application (Table 6). Likewise, in conjunction with the above, we observed the highest flower dry weight in the presence of a 30 ppm TiO_2_-NPs foliar application (Table 5). The toxicity of this substance at higher concentrations can be considered to have negative effects on some characters (like carotenoid, anthocyanin, and total phenol in this research)^38,65^.

The economic value of cut roses can be attributed to the length of the flower branch, flower diameter, and anthocyanin content^12,70^. Our experiments showed improvements in floral traits and anthocyanin content in roses under TiO_2_-NPs application and PRD treatments (Table 6). In a study carried out in the traditional way of deficit irrigation, the results showed a reduction in the length of the main branch and the diameter of the flower^49^. This report is consistent with our results, although in our experiment, PRD treatments had the best performance in floral traits (such as the number of flowers, flowering stem length, flower diameter, and flower tail diameter) compared to other treatments (Table 6). Another study has reported that applying TiO_2_-NPs results in a remarkable increase in biomass, flower number, leaf area, total chlorophyll, and carotenoid content in *Petunia* (*Petunia hybrida* L.) plants^69^. In our results, anthocyanin content increased significantly in PRD treatments. Still, there was no significant difference in FI and SDI treatments (Table 6), despite the results of Jafari et al. (2019)^63^ which reported an increase in anthocyanin in water deficiency conditions on the stock plant (*Matthiola incana* L.). This increase in anthocyanin could be due to the positive effect of TiO_2_-NPs on the plants reported in strawberry^71^ and rose (*Rosa damascena*)^70^.

As mentioned, deficit irrigation via the PRD method kept crop production stable and improved crop quality in critical economic parameters like flower number and steam length (Table 6). We have a significant increase in biomass WUE; the SDI treatment has a 51.3% lower outcome than PRD1 (Table 7). More importantly, we have a significant increase in WUE^1^. For practical purposes of water management in the greenhouse, we need to assess WUE^1^. The WUE does not show the correct result of water management performance due to not considering the leaching fraction. While the leaching fraction in artificial subtracts has been reported to be up to 50% in greenhouses^34^, Our results indicate that WUE^1^ in PRD treatments has significantly increased; PRD1 has a 50% and 23% increase in WUE^1^ compared to SDI and FI treatments, respectively (Table 7). Considering other water management approaches in greenhouses, as discussed above, the partial root-zone irrigation technique can be a feasible strategy with a low implementation cost in greenhouses with higher yield and water use efficiency^16,17, 72^; and even improve the quality of plants^18^.

## Conclusion

The experiment revealed a marginal advantage in certain biochemical characteristics with the application of PRD2 treatment instead of PRD1. Nevertheless, given the absence of noteworthy differences in vegetative traits, superior water use efficiency, and the convenience of executing the PRD1 irrigation approach in real-world settings, we advocate for adopting this irrigation method for hydroponic rose greenhouses in the commercial sector. More importantly, we could significantly increase WUE^1^ on an economical production scale by reducing 50% of irrigation volume, which probably reduces wastewater's refinement and release costs, diminishing environmental pollution. TiO_2_-NPs is suggested as a viable approach to alleviate the negative impacts of drought stress and enhance mineral absorption. Overall, the findings indicate that the PRD approach is superior, as it enhances growth attributes and water usage efficiency, resulting in heightened sustainability, decreased ecological effects of greenhouse hazardous wastewater, and overall profitability of the greenhouse.

## Data Availability

The data can be obtained by request and with the approval of the corresponding author.
